# Supplementary material for: Species Identification of Caviar Based on Multiple DNA Barcoding
Source: Molecules. 2023 Jun 28;28(13):5046. doi: 10.3390/molecules28135046 (PMC10343275; doi:10.3390/molecules28135046)
Supplement: Supplementary file 1 [file molecules-28-05046-s001.zip › molecules-2435469-supplementary.pdf]

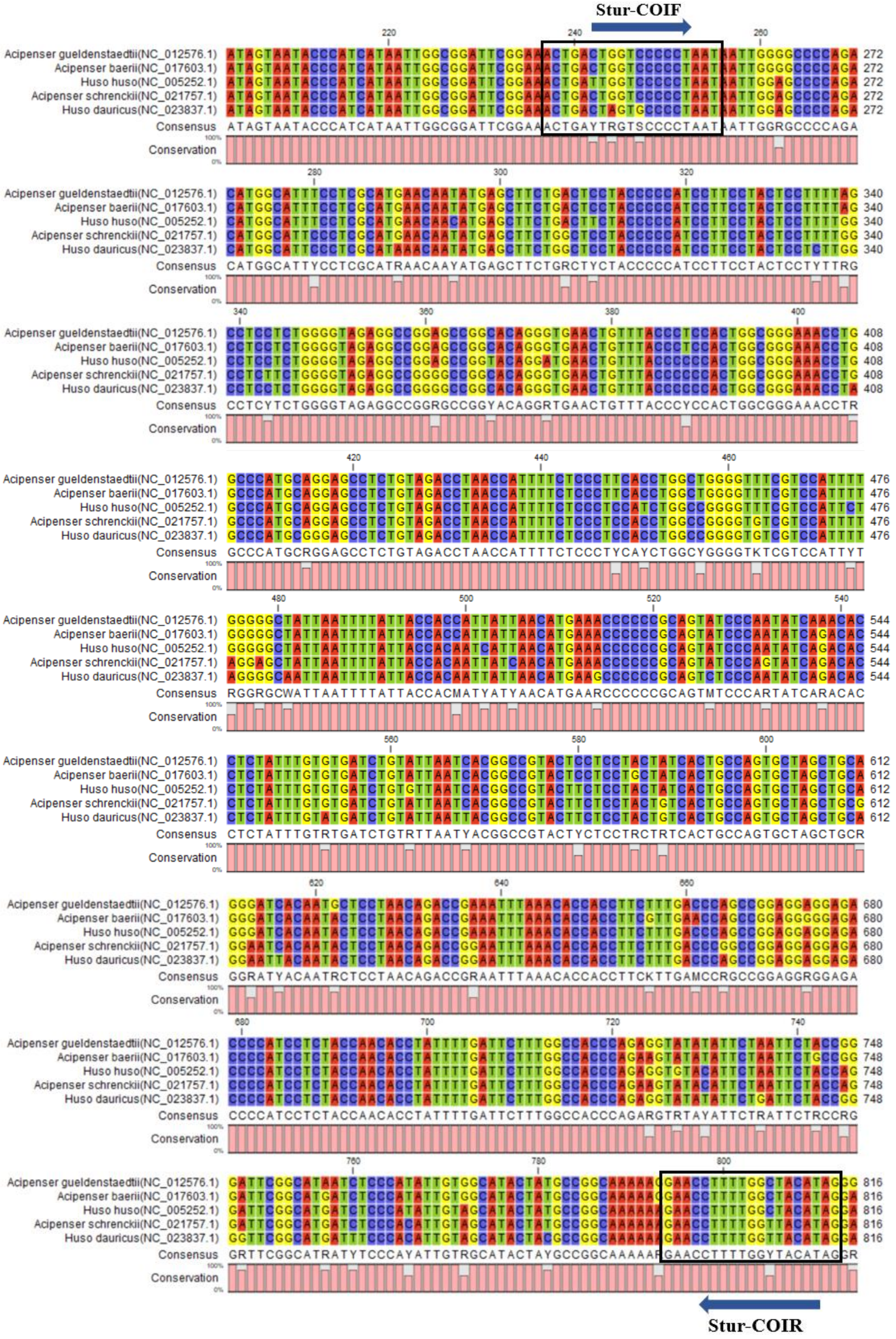
Stur-COIF
Acipenser gueldenstaedtii(NC_012576.1)
Acipenser baerii(NC_017603.1)
Huso huso(NC_005252.1)
Acipenser schrenckii(NC_021757.1)
Huso dauricus(NC_023837.1)
Consensus
Conservation
Stur-COIR

(a)

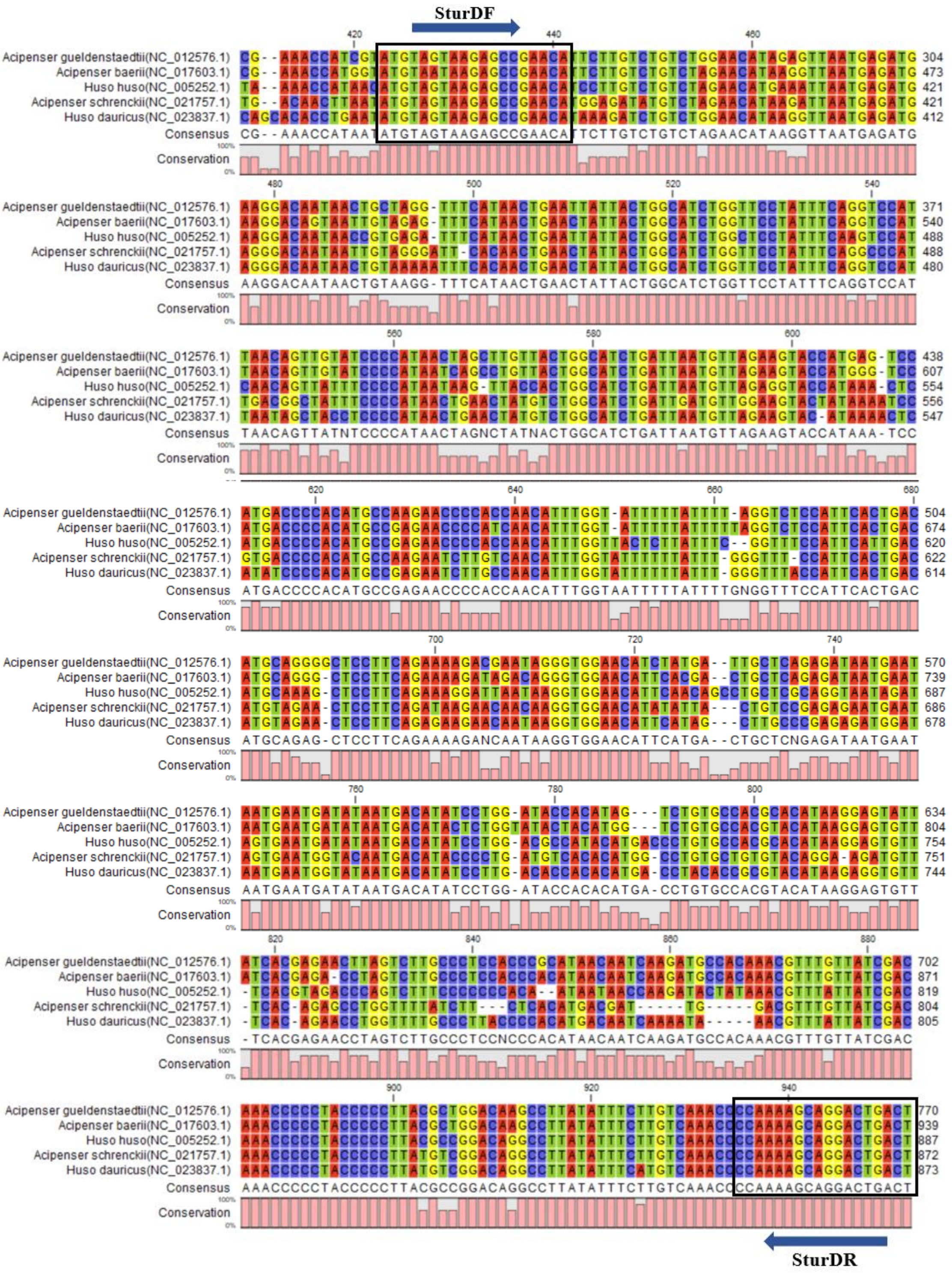

(b)

Figure S1. Sequence alignment of *COI* (a) and *D-loop* (b) primer designs. Using CLC Genomics Workbench 8.5, the *COI* and *D-loop* gene sequences of sturgeon were compared, and the conserved regions with significant differences in their sequences were selected to design primers.
